# ELID Polishing of Glass Substrates Using a Grainless Iron-Bonded Wheel with Free Abrasive Particles

**DOI:** 10.3390/mi16111226

**Published:** 2025-10-28

**Authors:** Huali Zhang, Xu Yan, Jicai Kuai, Dmitrii V. Ardashev

**Affiliations:** 1 School of Mechanical Engineering, Nantong Institute of Technology, Nantong 226001, China; 2 School of Mechanical Engineering, Nantong University, Nantong 226001, China; 3 School of Mechanical and Power Engineering, Henan Polytechnic University, Jiaozuo 454000, China; 4 Department of Mechanical Engineering, South Ural State University, Chelyabinsk 454080, Russia

**Keywords:** free abrasive particles, grainless iron-bonded wheel, glass substrate, ELID grinding, α-Fe_2_O_3_, surface quality, polishing efficiency

## Abstract

Conventional polishing of glass substrates often results in surface scratches caused by passivated abrasive particles, leading to defects and reduced yield. To overcome this limitation, an iron-bonded wheel with free abrasive grains was proposed in ELID (Electrolytic In-process Dressing) grinding. The polishing mechanisms were investigated using X-ray diffraction (XRD), Fourier transform infrared spectroscopy (FTIR), X-ray photoelectron spectroscopy (XPS), and micro-indentation. Polishing efficiency was assessed via mass loss measurements, surface quality was characterized by atomic force microscopy (AFM), and optical transmittance was evaluated using a transmittance meter. Results indicate that the proposed wheel does not contain fixed abrasive particles but generates α-Fe_2_O_3_ particles during polishing, effectively preventing surface scratches and achieving superior surface quality. The polishing efficiency ranged from 0.02 to 1.6 μm/min, with a resulting surface roughness of 2.1 nm. Furthermore, the glass substrates exhibited higher transmittance compared to those polished using conventional methods, contributing to improved display performance and brightness. This polishing technology demonstrates significant potential for applications in the display industry.

## 1. Introduction

Glass substrates are essential components in liquid crystal display (LCD) televisions, tablet computers, mobile phones, and other electronic products. For many years, their production has been dominated by the United States and Japan. Since 2011, Chinese enterprises have introduced foreign technologies and equipment, enabling the manufacture of lower-generation glass substrates (below the 6th generation, approximately 1500 × 1850 mm). In contrast, foreign manufacturers have achieved production of higher-generation substrates exceeding the 10th generation (approximately 2880 × 3130 mm). Significant gaps, however, remain in terms of quality, efficiency, and yield between domestic and foreign products [1]. In addition to display applications, glass substrates offer excellent electrical and mechanical properties and superior thermal stability. These features allow them to surpass the limitations of traditional organic substrates, making them highly promising for use in high-performance computing and AI chip packaging [2].

High surface accuracy, low roughness, and excellent optical transmittance are critical requirements for glass substrates. Currently, domestic yields are limited by micron-level scratches and other defects introduced during polishing. These defects can cause broken data and scan lines in LCD panels, resulting in image blurring, distortion, and other display issues. The origin of these scratches is often attributed to contact with hard mechanical components during processing and transport [3]. A broader perspective holds that the use of rare earth polishing powders such as cerium oxide (CeO_2_) and iron oxide (α-Fe_2_O_3_) offers high polishing efficiency and low cost, yet coarse abrasive particles and excessive polishing pressure remain the primary contributing factors [4,5]. Others suggest that uneven abrasive dust, impurity particles, and residual grinding powders contribute to surface defects [6]. Various solutions have been explored, including improving powder uniformity, removing coarse particles, reducing polishing pressure, and using micro-porous polishing pads [7], with some success. Magnetorheological polishing has also been effective in scratch reduction [8]; however, it is complex, introduces surface contaminants (e.g., Fe, Ce), has low material removal rates, and requires costly equipment, limiting its practical application [9]. Ion beam polishing yields the finest surface quality without damage, but it is extremely inefficient and prohibitively expensive. More recently, electrolytic in-process dressing (ELID) grinding has been applied to achieve ultra-precision surfaces on glass substrates, with nanoscale accuracy reported [10,11,12,13]. Nevertheless, detached passivated particles in the grinding zone still cause micron-level scratches. Therefore, an effective method to fully eliminate scratches remains urgently needed.

To address the persistent issue of surface scratches, this study proposes an iron-based grinding wheel without embedded abrasive grains (grainless). It demonstrates significant advantages in terms of processing efficiency, achieving surface quality comparable to chemical mechanical polishing. Its primary constituents comprise approximately 80–85% α-iron powder, 5–10% copper powder, 1–3% tin powder, 0.5–1.5% graphite, with minor quantities of other trace elements. During electrolytic in-process dressing (ELID), an oxide film composed of various iron oxides forms on the wheel surface. Under the high temperatures in the grinding zone, this film undergoes phase transformation, generating free α-Fe_2_O_3_ abrasive particles in situ [14,15]. These particles act as highly effective polishing agents for glass substrates and optical components, enabling high-quality finishing without introducing fixed abrasives.

In this study, the polishing mechanism of the grainless iron-based wheel generating free α-Fe_2_O_3_ abrasive particles was investigated. The formation mechanism of α-Fe_2_O_3_ was analyzed using XRD, FTIR, and XPS. The mechanical properties of the oxide film were characterized using micro-indentation. Based on these analyses, the surface quality, material removal rate, and optical transmittance of glass substrates polished with this novel wheel were systematically evaluated.

## 2. Polishing Principle of Grainless Iron Grinding Wheel

The grainless iron-based grinding wheel, which does not incorporate abrasive particles, is inherently unsuitable for achieving high surface quality through conventional abrasive cutting. During the ELID electrolysis process, however, an oxide film forms on the wheel surface, comprising a substantial amount of iron oxides. Under the elevated temperatures present in the grinding zone, these oxides are transformed into α-Fe_2_O_3_ particles. As α-Fe_2_O_3_ exhibits polishing performance second only to CeO_2_, the grainless iron-based grinding wheel thereby attains effective polishing capability. The principle of the corresponding electrochemical reaction is outlined as follows:

(1) $$Fe\to Fe^{+2}+2e^{-}$$

(2) $$Fe^{+2}\to Fe^{+3}+e^{-}$$

(3) $$H_{2}O\to H^{+}+OH^{-}$$

(4) $$Fe^{2+}+2OH^{-}\to Fe(OH{)}_{2}$$

(5) $$Fe^{3+}+3OH^{-}\to Fe(OH{)}_{3}$$

The Fe(OH)_3_ formed on the surface of the grinding wheel can also be represented as Fe_2_O_3_·3H_2_O. During the grinding process, the precipitated Fe(OH)_3_ undergoes dehydration and structural transformation under the influence of elevated grinding temperatures. The hydrated iron oxide, Fe_2_O_3_·3H_2_O, serves only as a transient intermediate, as the removal of lattice water is rapidly completed with increasing temperature:

(6) $$Fe_{2}O_{3}\cdot 3H_{2}O⟶Fe_{2}O_{3}+3H_{2}O$$

During grinding, substantial heat accumulates in the grinding zone, producing elevated temperatures. At these temperatures, adsorbed and lattice water in the oxide film are first removed. Subsequently, γ-Fe_2_O_3_ forms at approximately 300 °C, and with further heating to 600 °C, γ-Fe_2_O_3_ transforms into α-Fe_2_O_3_, as expressed in formula (7).

(7) $$\gamma \text{-}{\mathrm{Fe}}_{2}O_{3}\overset{\Delta }{\to }\alpha \text{-}{\mathrm{Fe}}_{2}O_{3}$$

## 3. Experimental Equipment and Methods

Polishing experiments were performed on a surface grinding machine, the MM7140 (Hangzhou Machine Tool Factory, Hangzhou, China), equipped with ELID apparatus and a micro-feed table, using a grainless iron-based grinding wheel on glass substrates. The grinding wheel has undergone pre-treatment by the manufacturer prior to dispatch, achieving the surface condition required for direct ELID electrolytic processing. The ELID electrolytic and polishing parameters are summarized in Table 1.

Prior to experimentation, the grainless iron-based grinding wheel (W0) and the grainless CeO_2_ grinding wheel (W1) were shaped by EDM and subsequently subjected to ELID electrolysis. The oxide films produced during electrolysis were characterized using X-ray diffraction (XRD, D8 Advance, Bruker, Germany), Fourier transform infrared spectroscopy (FTIR, Magna-IR750, Nicolet, Madison, WI, USA), and X-ray photoelectron spectroscopy (XPS, ESCALAB 250XI, Thermo Fisher, Waltham, MA, USA). The mechanical properties of the grinding wheels, including hardness, elastic modulus, and stiffness, were evaluated using a micro-indentation tester (MHT, Anton Paar, Graz, Austria).

**Figure 1:**
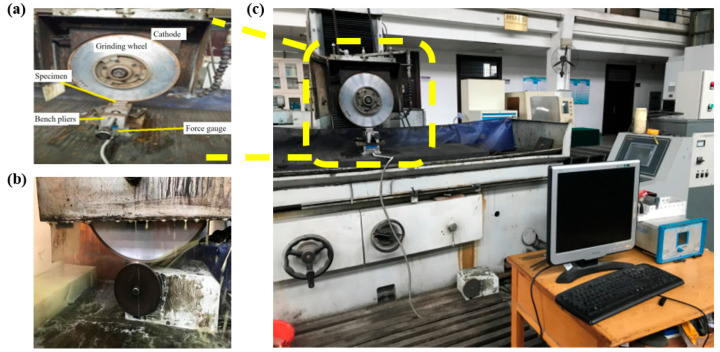
ELID grainless iron-based grinding wheel polishing equipment. (**a**) ELID sharpening system, (**b**) EDM shaping system, (**c**) overall of the ELID polishing setup.

In the polishing procedure, the glass substrates were initially coarse-polished with the W1 CeO_2_ grinding wheel, followed by fine and super polishing with the W0 grainless iron-based wheel. The samples used in the experiment were sodium-calcium glass, with specific dimensions of 20 mm × 20 mm × 1 mm (length × width × thickness) and a density of 2.5 g/cm^3^. Surface morphology was examined with an atomic force microscope (AFM, Multimode VIII, Veeco, Jersey City, NJ, USA), and optical transmittance was measured with a transmission meter (DR81, Dongru Electronic Technology Co., Ltd., Guangzhou, China). The polishing equipment is illustrated in Figure 1.

## 4. Results and Discussion

### 4.1. Polishing Surface Quality

**Figure 2:**
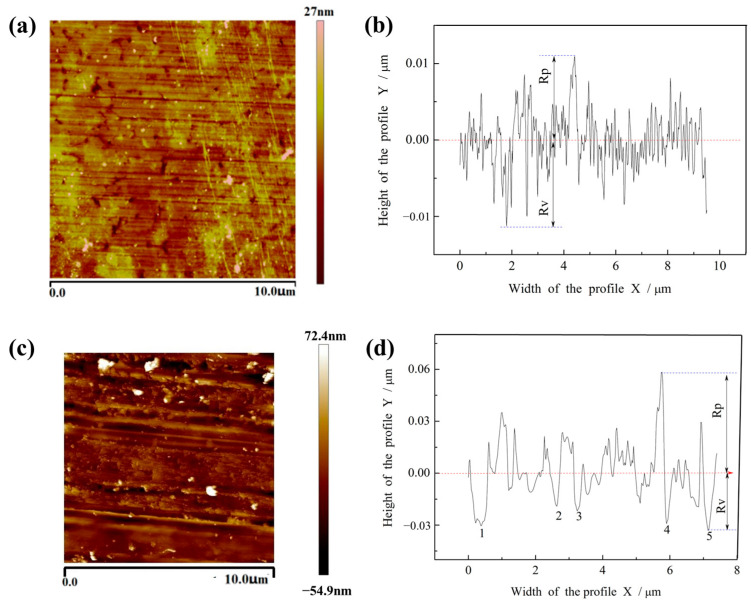
AFM test results of glass substrate. (**a**) Grainless iron-based grinding wheel polishing glass substrate, A 10 μm × 10 μm area with a resolution of 0.1−0.2 nm, Five replicate measurements, (**b**) Figure 2a Section profile analysis, (**c**) Polishing glass substrate with abrasive iron base grinding wheel, A 10 μm × 10 μm area with a resolution of 0.1−0.2 nm, Five replicate measurements, (**d**) Figure 2c Section profile analysis.

Figure 2a shows the AFM results of glass substrates polished with the grainless iron-based grinding wheel (W0). The surface roughness Ra of the glass substrate prior to polishing is 100 nm. Several nanoscale plastic scratches with clear orientation and directivity can be observed, which are attributed to the cutting action of α-Fe_2_O_3_ particles. In addition, a few nano-pits are visible, likely originating from defects in the glass substrate itself. The corresponding cross-sectional profile (Figure 2b) confirms that the surface exhibits no coarse scratches and maintains high smoothness. By contrast, Figure 2c displays the AFM results for polishing with the conventional CeO_2_ grinding wheel (W1). In this case, plastic scratches are more uniformly distributed, but several deep, coarse, and laterally oriented scratches are clearly present. The cross-sectional profile in Figure 2d indicates significant surface damage, with some scratches exceeding 30 nm in depth, which are difficult to remove in subsequent processes and thus contribute to low substrate yield and increased production costs.

The quantitative roughness parameters of the polished surfaces are summarized in Table 2. Compared with W1, the W0 grinding wheel achieves significantly lower values of Ra, Rz, Rp, and Rv, confirming its superior surface quality. It also outperforms C. Rameshkumar nanoparticles (CeO_2_) as an abrasive in chemical mechanical polishing in terms of surface roughness [16].

The improved performance of W0 can be attributed to the in situ generation of nanoscale α-Fe_2_O_3_ particles during polishing. Owing to their lower hardness compared with CeO_2_ particles, the polishing action is comparatively gentle, resulting in fewer scratches and higher surface accuracy. These findings demonstrate that the W0 grinding wheel provides distinct advantages in reducing surface defects and enabling ultra-precision polishing of glass substrates.

### 4.2. Polishing Removal Rate

For conventional micro-powder grinding wheels, a reduction in particle size increases the specific surface area and surface energy, which promotes agglomeration. Consequently, the effective abrasive distribution density is generally below 12.5%. In contrast, the grainless iron-based grinding wheel (W0) eliminates abrasive agglomeration, enabling an effective abrasive density exceeding 80%. This structural advantage results in a significantly higher material removal rate. The experiment employed a material removal rate characterisation method based on thickness monitoring, calculating the removal rate by measuring the thickness change of glass specimens before and after polishing.

**Figure 3:**
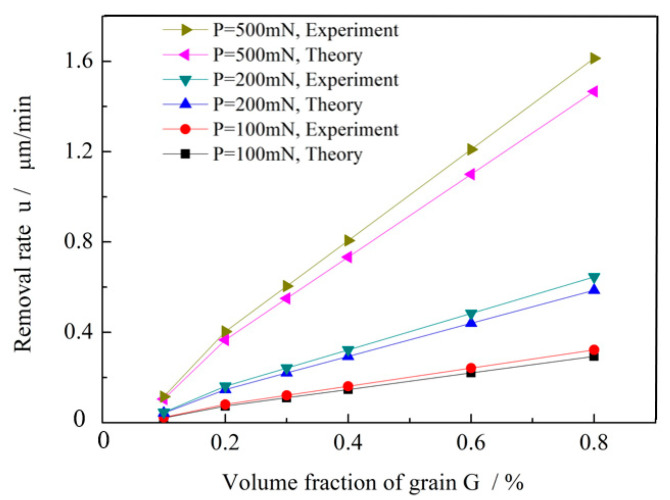
Removal rates of glass substrate materials.

Figure 3 shows the material removal rate of glass substrates polished with W0 as a function of abrasive particle volume fraction and polishing pressure. The removal rate increases sharply as the particle volume fraction rises from 0.1 to 0.8 and the polishing pressure increases from 100 to 500 mN. Conforms to the principles of the Adaptive Shear Gradient Thickening Polishing (AS-GTP) material removal rate model established by Li M [17]. Under optimal conditions (500 mN pressure and 80% abrasive volume fraction), the maximum removal rate reaches 1.6 μm/min.

A comparison with the conventional polishing method is summarized in Table 3. The results indicate that the removal rate of W0 is nearly four times higher than that of the CeO_2_-based grinding wheel, highlighting the superior efficiency of the grainless iron-based wheel.

### 4.3. Optical Transmittance

**Figure 4:**
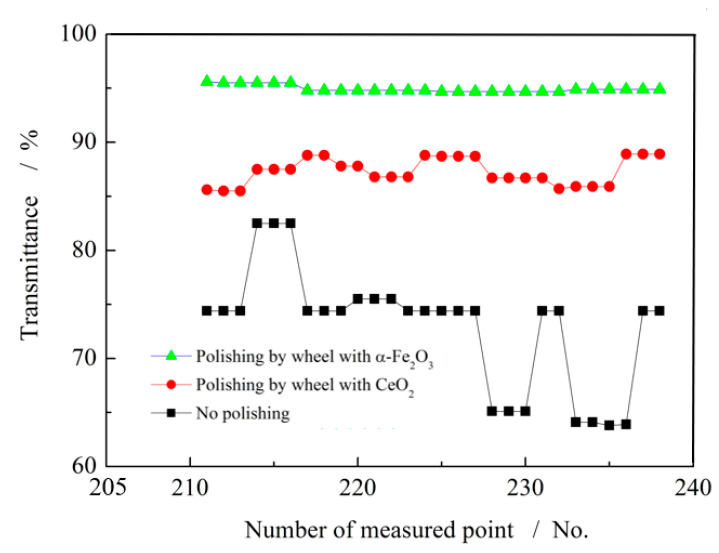
Test results of light transmittance of glass substrate.

Optical transmittance directly affects the image quality and brightness of display screens, making it a critical performance index for glass substrates. The transmittance of glass substrates was measured before and after superfine polishing with the CeO_2_ grinding wheel and the grainless iron-based grinding wheel (W0), using a transmission meter. The results are presented in Figure 4.

Prior to polishing, the transmittance of the substrates ranged from 65% to 82%. After polishing with the CeO_2_ grinding wheel, the transmittance improved to 85–90%. Following polishing with the grainless iron-based grinding wheel, the transmittance further increased to 93–95%. It also outperforms the transmittance achieved by Jee-Hun Maeng’s chemical polishing process [19]. These results demonstrate that the W0 grinding wheel provides superior optical performance and is particularly suitable for the ultra-precision polishing of glass substrates.

### 4.4. Thermal Effects and Oxide Film Transformation

Previous studies have reported the high thermal loads involved in precision grinding. Theoretically, the temperature during the stages of single abrasive friction, ploughing, and grinding may reach as high as 1085 °C in the friction stage [20]. Malkin [21] also suggested that in precision grinding, slippage between abrasive particles and the workpiece can elevate the grinding temperature to near the melting point of the material. The polishing temperature in ELID processes has been studied in detail, with reported values ranging from 200 to 500 °C, and maximum grinding temperatures reaching 559.83–573.92 °C [22].

In related work, the temperature-sensitive characteristics and distribution of α-Fe_2_O_3_ particles around abrasive grains have been examined, leading to the proposal of a layered oxide film structure. In this model, the temperature is highest adjacent to the abrasive grain, where α-Fe_2_O_3_ dominates, while lower-temperature regions farther from the grain consist primarily of γ-Fe_2_O_3_, FeOOH, and Fe(OH)_3_ [23]. These findings indicate that the thermal conditions in the ELID polishing zone are sufficient for the generation and transformation of α-Fe_2_O_3_ particles.

**Figure 5:**
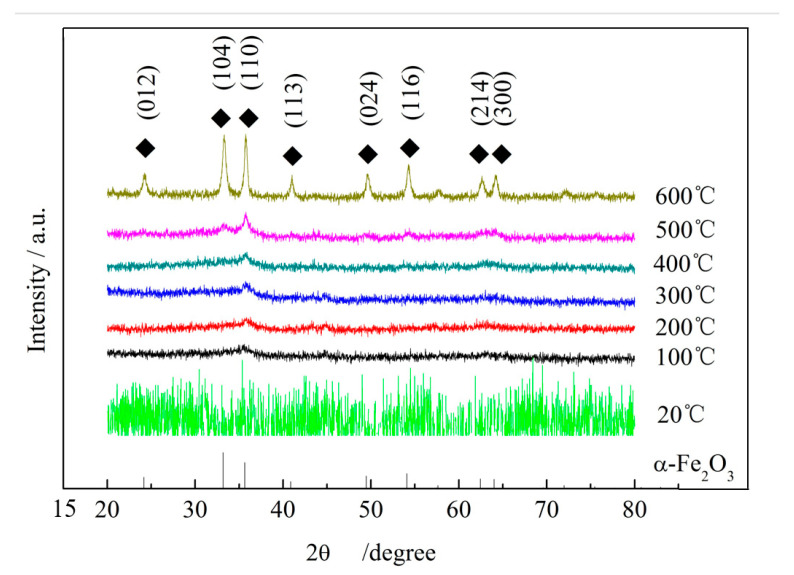
XRD test results of the oxide film of grainless iron-based grinding wheel.

Figure 5 presents the XRD spectra of the oxide film on the grinding wheel surface under variable temperatures (100–600 °C). At 100 °C, the 104 and 110 peaks and the 214 and 300 peaks of α-Fe_2_O_3_ overlap, with other peaks not yet visible. As the temperature increases, additional diffraction peaks appear progressively. At 300 °C, peaks 104 and 110 are fully resolved; at 600 °C, peaks 214 and 300 also emerge clearly, along with the remaining peaks at 012, 113, 024, and 116. These positions correspond to the PDF standard card 33-0664, confirming the crystalline identity of α-Fe_2_O_3_. Overall, the gradual emergence of these diffraction peaks as the temperature rises from 100 °C to 600 °C demonstrates the progressive generation and transformation of α-Fe_2_O_3_ particles.

### 4.5. Comparative Analysis of Abrasive Density and Oxide Film Composition

Traditional abrasive grinding wheels are sintered under high temperature and pressure. To avoid particle agglomeration, the abrasive density is intentionally kept low, typically not exceeding 12.5% for CeO_2_ wheels (W1). In contrast, the grainless iron-based grinding wheel (W0) generates α-Fe_2_O_3_ particles in situ through the thermal transformation of iron oxides within the oxide film. This process eliminates agglomeration issues and allows α-Fe_2_O_3_ particles to be densely distributed across the grinding wheel surface. If the conversion rate approaches 100%, the abrasive density of α-Fe_2_O_3_ particles can reach up to 80%, thereby enabling a high material removal rate. The determination method is calculated using the abrasive rate formula (8) from grinding theory.

(8) ρ=(Np·Vp)/(Ac·h)

In the equation, Np denotes the number density of protruding edges, Vp represents the volume of a single abrasive grain, Ac indicates the contact area, and h denotes the cutting depth.

**Figure 6:**
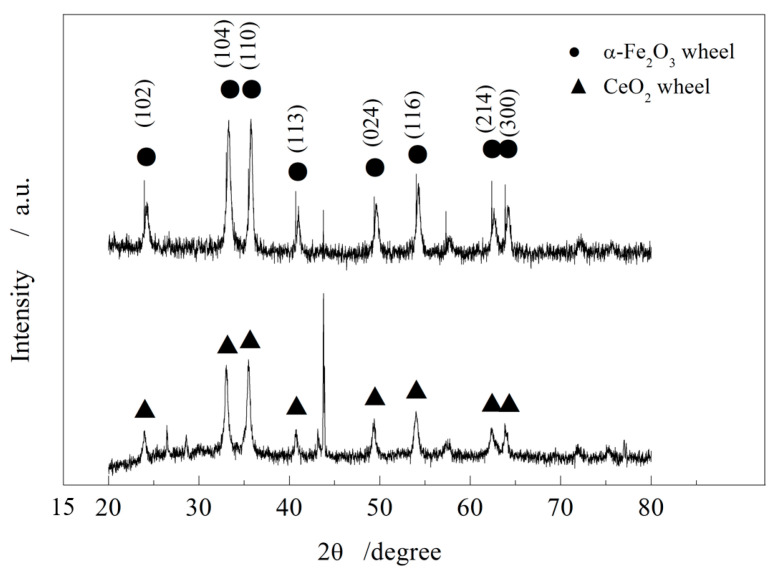
Test comparison between non-abrasive and abrasive iron-based grinding wheels.

Figure 6 presents the XRD spectra of oxide films from W1 and W0. The characteristic peak intensities of W0 are approximately 1–2 times higher than those of W1, indicating that the content of α-Fe_2_O_3_ particles on the W0 surface is significantly greater. This higher abrasive density directly translates into superior polishing efficiency, making W0 more suitable for ultra-fine polishing of optical materials such as glass substrates.

**Figure 7:**
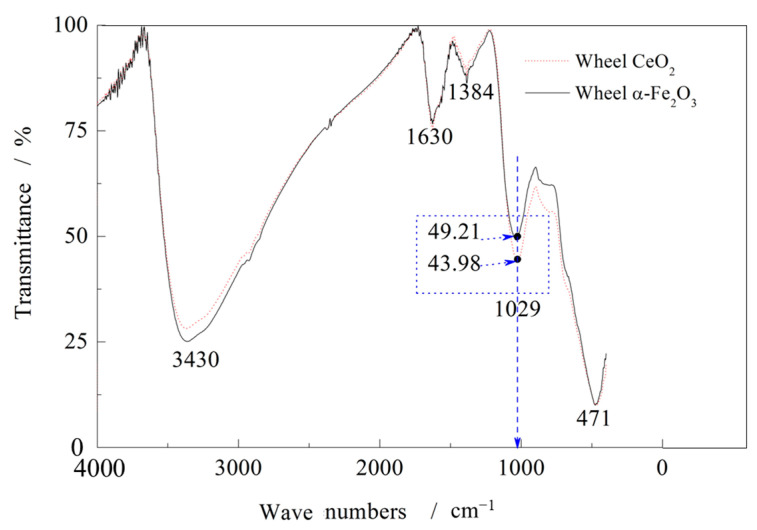
Infrared spectrum test of wheel oxide film. (Background correction has been applied. The horizontal axis in the figure represents the measured wavenumber range.).

The infrared spectra of the two wheels are compared in Figure 7. The absorption band at 1029 cm^−1^ corresponds to the Fe–O vibration of α-Fe_2_O_3_, confirming its presence in both oxide films. However, the stronger intensity observed for W0 demonstrates a higher α-Fe_2_O_3_ content relative to W1.

**Figure 8:**
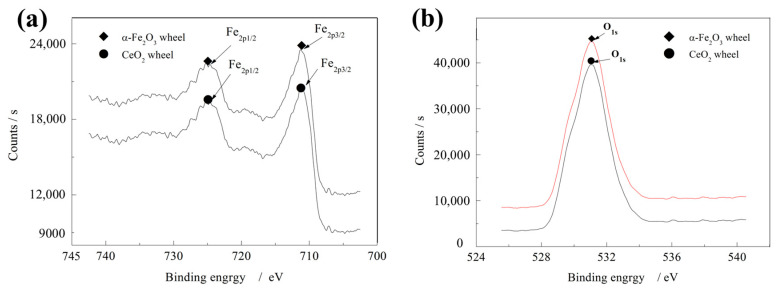
XPS test of wheel oxide film. (**a**) Fe^3+^ content XPS test, (**b**) XPS test of O element content.

Further confirmation is provided by XPS analysis (Figure 8). The Fe 2p spectrum (Figure 8a) exhibits binding energies at 710.6 eV (Fe 2p_3_/_2_) and 724.3 eV (Fe 2p_1_/_2_), with a peak separation of 13.7 eV, consistent with the standard spectrum of Fe^3+^ in α-Fe_2_O_3_ [24,25]. The oxygen content analysis (Figure 8b) shows that the oxide film of W0 contains a higher proportion of iron oxides compared with W1, corroborating the XRD and FTIR results.

These findings confirm that the grainless iron-based grinding wheel generates a higher density of α-Fe_2_O_3_ particles than conventional CeO_2_ wheels. In conventional abrasive grinding wheels, abrasive particles are the primary cutting agents, while the oxide film in ELID wheels provides supplementary polishing. In the case of W0, however, the fine polishing effect is derived predominantly from the α-Fe_2_O_3_ particles embedded in the oxide film. As a result, ELID grinding with W0 enables superior surface quality in the ultra-precision polishing of glass substrates.

### 4.6. Analysis of Mechanical Properties of Grainless Iron-Based Grinding Wheel

**Figure 9:**
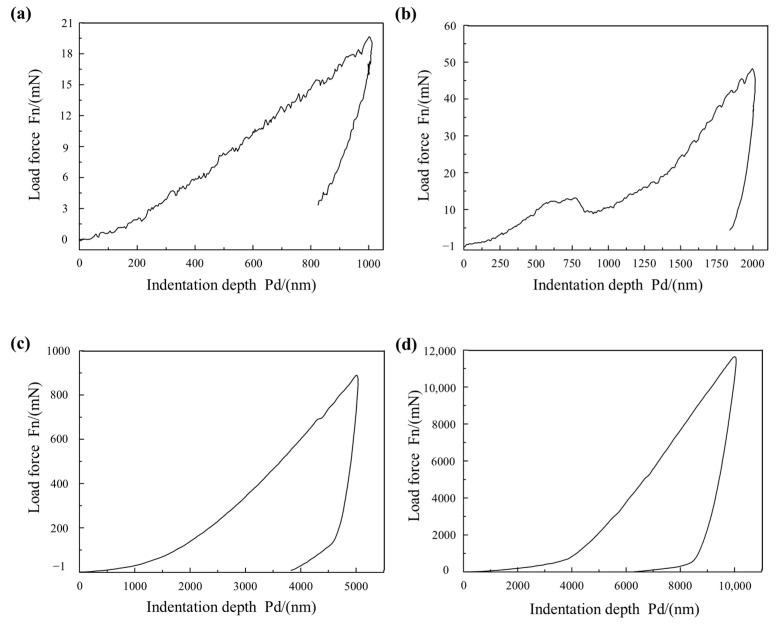
Load displacement curve. (**a**) Indentation 0.001 mm load displacement curve, (**b**) Indentation 0.002 mm load displacement curve, (**c**) Indentation 0.005 mm load displacement curve, (**d**) Indentation 0.01 mm load displacement curve.

**Figure 10:**
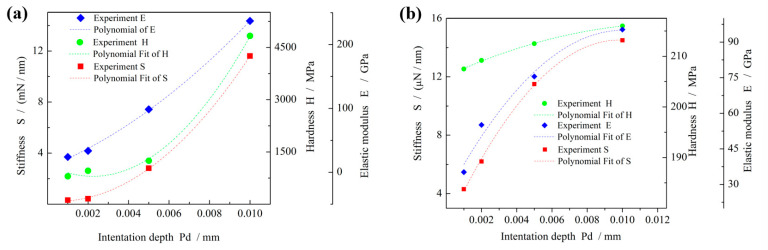
Changes of mechanical properties. (**a**) Mechanical properties of grainless iron-based grinding wheel, (**b**) Mechanical properties of abrasive iron-based grinding wheel.

The mechanical behavior of the oxide film on the grainless iron-based grinding wheel (W0) was evaluated by nano-indentation testing. The load–displacement curves are shown in Figure 9, while the variation of mechanical properties with indentation depth is presented in Figure 10 [26]. All experiments were conducted at a loading rate of 100 mN/min, with a dwell time of 5 s, and measurements were taken at room temperature (25 °C).

At an indentation depth of 0.001 mm (Figure 9a), the displacement increases nonlinearly with load, and creep is observed along nearly the entire curve. This behavior is attributed to the collapse of pores within the oxide film under the action of the indenter. When the indentation depth increases to 0.002 mm (Figure 9b), the displacement rises while the load decreases, reflecting large-scale pore collapse in the oxide film. At greater depths of 0.005 mm and 0.01 mm (Figure 9c,d), the curves become smooth, and no obvious collapse is observed. At these depths, the load increases markedly to 1000–1200 mN, indicating that the indenter has reached the grinding wheel substrate.

The derived mechanical properties are shown in Figure 10a. Hardness (H), elastic modulus (E), and stiffness (S) all increase with indentation depth. Specifically, hardness increases from 800 to 4900 MPa, elastic modulus from 23 to 230 GPa, and stiffness from 0.3 to 11 mN/nm. At shallow depths, the measured values primarily reflect the properties of the oxide film, where the matrix influence is minimal. With increasing indentation depth, the contribution of the grinding wheel substrate becomes more significant, resulting in higher measured values. At the maximum indentation depth of 0.01 mm, the measured hardness, elastic modulus, and stiffness approach those of the grinding wheel substrate.

The intrinsic mechanical properties of the oxide film can therefore be estimated as in Table 4.

Previous studies have reported significantly lower stiffness values for oxide films generated on conventional iron-based grinding wheels during ELID electrolysis. For example, the stiffness of oxide films on gray iron-based grinding wheels was found to vary from 27 to 95 μN/nm [27], as shown in Figure 10b. Similarly, Fathima [28] measured a stiffness of 16.34 μN/nm, which is of the same order of magnitude. By comparison, the stiffness of oxide films formed on the grainless iron-based grinding wheel (W0) is substantially higher. This difference can be attributed to variations in the electrolysis process. In traditional abrasive grinding wheels, the presence of abrasive particles causes non-uniform electric field distribution during ELID electrolysis. As a result, the generated oxide film is weak, discontinuous, and unevenly distributed. In contrast, for the grainless iron-based wheel, the electric field is uniform, leading to the formation of a continuous oxide film with more consistent mechanical properties [11].

The oxide film can be considered analogous to a spring. During ELID polishing, α-Fe_2_O_3_ particles embedded in the oxide film are pressed against the workpiece surface with a spring force, enabling material removal. In W0, the oxide film is evenly distributed, allowing α-Fe_2_O_3_ particles to act uniformly across the workpiece surface. This uniform action ensures consistent material removal and superior polishing performance compared with conventional iron-based grinding wheels.

## 5. Conclusions

In this study, the polishing performance and material characteristics of a grainless iron-based grinding wheel (W0) were systematically investigated and compared with those of a conventional CeO_2_ grinding wheel (W1). The main conclusions are as follows:

(1) Polishing mechanism: High grinding temperatures during ELID electrolysis promote the transformation of iron oxides in the oxide film into α-Fe_2_O_3_ particles. The collaborative cutting action of these densely distributed nanoscale particles enables ultra-smooth surface accuracy.
(2) Polishing efficiency: In super-fine polishing at low pressures, the process achieves high precision but low efficiency. With increasing polishing pressure, the material removal rate rises rapidly, reaching a maximum of 1.6 μm/min—nearly four times higher than that of the conventional CeO_2_ grinding wheel.
(3) Surface quality: AFM analysis revealed that W0 achieves a surface roughness of Ra ≈ 2.1 nm, with no visible scratches or surface defects. This confirms the capability of W0 to produce ultra-precision surfaces.
(4) Optical transmittance: After polishing with W0, glass substrates exhibited transmittance of 93–95%, approximately 5% higher than that obtained using CeO_2_ wheels. This improvement indicates superior potential for applications in display technologies, where enhanced image clarity and brightness are critical.

Overall, the grainless iron-based grinding wheel exhibits clear advantages over conventional CeO_2_ grinding wheels in terms of surface quality, material removal efficiency, optical transmittance, and oxide film performance. The in situ generation of α-Fe_2_O_3_ particles and the uniform distribution of the oxide film are the primary factors contributing to these improvements. These findings demonstrate that W0 is highly suitable for ultra-precision polishing of optical materials, particularly glass substrates, and provide a theoretical and experimental basis for its further application in advanced optical manufacturing. Concurrently, the polishing mechanism proposed in this study—based on the in situ electrolytic interaction between ELID and iron-based binders—can be extended to typical brittle material substrates such as silicon, silicon dioxide, calcium fluoride, and sapphire. This approach holds broad application prospects in the fields of integrated circuits, optoelectronics, and high-end optical components.

## Figures and Tables

**Table 1:** ELID electrolytic parameters Polishing parameters.

Voltage U/V	Electric Current I/A	Pulse Width T_on_/μs	Interpulse Width T_off_ /μs	Duty Cycle	Grinding Wheel Speed V_s_/m/s	Feed Rate Vw /m/min	Polishing Depth ap /μm	Pre-Electrolysis Time t/min	Electrolyte pH Value	Circulation Method	Electrolyte Ratio	Grinding Wheel Type
60, 90, 120	50−2	99−1	99−1	50%	15.7	0.2−0.4	1−0.01	30−60	8−9	pump circulation	1:50	W1 W0

**Table 2:** Surface roughness parameters of glass substrates polished by W0 and W1 wheels. (Take the mean of five repeated measurements, with a coefficient of variation of 8%.).

Grinding Wheel	Ra (nm)	Rz (nm)	Rp (nm)	Rv (nm)
W0 (Fe-based, grainless)	2.1	12.2	6.99	11.56
W1 (CeO_2_, conventional)	9.4	62.1	42.1	38.5

**Table 3:** Comparison of material removal rates between W0 and conventional polishing methods.

Polishing Method	Polishing Conditions	Removal Rate (μm/min)	Relative Efficiency
W0(Fe-based, grainless)	500 mN, 80% volume fraction, 1000 r/min, 10–30min	1.6	~4× higher
W1(CeO_2_, conventional)	Standard conditions [18]	0.409	Baseline

**Table 4:** Mechanical properties of the oxide film and grinding wheel substrate.

Material	Hardness H (MPa)	Elastic Modulus E (GPa)	Stiffness S (mN/nm)
Oxide film (intrinsic)	700~900	21~25	0.2~0.4
Grinding wheel substrate	4800~5000	220~240	10~12

## Data Availability

Data is contained within the article.
